# Comprehensive analysis of a cuproptosis-related ceRNA network implicates a potential endocrine therapy resistance mechanism in ER-positive breast cancer

**DOI:** 10.1186/s12920-023-01511-0

**Published:** 2023-05-05

**Authors:** Dongni Zhang, Wenping Lu, Zhili Zhuo, Yanan Wang, Weixuan Zhang, Mengfan Zhang

**Affiliations:** Oncology Department, China Academy of Chinese Medical Sciences Guang’anmen Hospital, Beijing, China

**Keywords:** Breast cancer, ceRNA network, Endocrine therapy resistance, Cuproptosis, Prognosis

## Abstract

**Background:**

While adjuvant endocrine therapy (ET) may decrease the mortality rate of estrogen receptor-positive (ER+) breast cancer (BC), the likelihood of relapse and metastasis due to ET resistance remains high. Cuproptosis is a recently discovered regulated cell death (RCD), whose role in tumors has yet to be elucidated. Thus, there is a need to study its specific regulatory mechanism in resistance to ET in BC, to identify novel therapeutic targets.

**Methods:**

The prognostic cuproptosis-related genes (CRGs) in ER+ BC were filtered by undergoing Cox regression and least absolute shrinkage and selection operator (LASSO) regression analyses in TCGA-BRCA, and a CRGs risk signature was constructed using the correlation coefficient. Immune infiltration analysis, immune function analysis, tumor microenvironment (TME) analysis, immune checkpoint analysis, immunotherapy response analysis, drug sensitivity analysis, and pathway activation analysis were carried out among the high- and low-risk groups in turn. The central CRG of cuproptosis in ER+ BC resistance to ET was acquired through the intersection of protein interaction network (PPI) analysis, genes differentially expressed (DEGs) between human BC cells LCC9 and MCF-7 (GSE159968), and CRGs with prognostic significance in TCGA-BRCA ER+ BC. The miRNAs upstream of the core CRGs were predicted based on the intersection of 4 databases, miRDB, RNA22, miRWalk, and RNAlnter. Candidate miRNAs consisted of the intersection of predicted miRNAs and miRNAs differentially expressed in the LCC9 and MCF-7 cell lines (GSE159979). Candidate lncRNAs were the intersection of the differential lncRNAs from the LCC9 and MCF-7 cell lines and the survival-related lncRNAs obtained from a univariate Cox regression analysis. Pearson's correlation analysis was performed between mRNA-miRNA, miRNA-lncRNA, and mRNA-lncRNA expression separately.

**Results:**

We constructed A risk signature of 4-CRGs to predict the prognosis of ER+ BC in TCGA-BRCA, a risk score = DLD*0.378 + DBT*0.201 + DLAT*0.380 + ATP7A*0.447 was used as the definition of the formula. There were significant differences between the high- and low-risk groups based on the risk score of 4-CRGs in aspects of immune infiltration, immune function, expression levels of immune checkpoint genes, and signaling pathways. *DLD* was determined to be the central CRG of cuproptosis in ER+ BC resistance to ET through the intersection of the PPI network analysis, DEGs between LCC9 and MCF-7 and 4-CRGs. Two miRNAs hsa-miR-370-3p and hsa-miR-432-5p were found taking *DLD* mRNA as a target, and the lncRNA C6orf99 has been hypothesized to be a competitive endogenous RNA that regulates *DLD* mRNA expression by sponging off hsa-miR-370-3p and hsa-miR-432-5p.

**Conclusion:**

This study built a prognostic model based on genes related to cuproptosis in ER+ BC. We considered *DLD* to be the core gene associated with resistance to ET in ER+ BC via copper metabolism. The search for promising therapeutic targets led to the establishment of a cuproptosis-related ceRNA network C6orf99/hsa-miR-370-3p and hsa-miR-432-5p/*DLD*.

**Supplementary Information:**

The online version contains supplementary material available at 10.1186/s12920-023-01511-0.

## Introduction

The incidence of female breast cancer (BC) has continued to rise since the 1970s and has become one of the leading causes of global cancer morbidity rates worldwide [1]. BCs have high heterogeneity with multiple subtypes, with incidence and recurrence rates varying widely depending on the molecular profile [2]. BCs that are estrogen receptor-positive (ER+) comprise approximately 80% of BC patients in the clinic, which is the most prevalent subtype and has an estrogen dependence for growth [3]. For ER+ BC patients, endocrine therapies (ET) including selective estrogen receptor down-regulators (SERDs), aromatase inhibitors (AIs), and selective estrogen receptor modulators (SERMs) are critical, among which tamoxifen (TAM) is a mainstay of treatment in use [4]. Even though adjuvant TAM treatment can reduce the mortality rate of ER+ BC by 31% [5], relapse and metastasis due to TAM resistance are still present in 30–50% of patients [6], and this has severely affected their survival and quality of life. Given the complex involvement of multiple signaling pathways and the fragmented understanding of drug resistance mechanisms, although there is substantial research into the pathways leading to resistance to ET in BC and drugs of adaptive mechanisms have entered the clinic, new resistance invariably develops. Consequently, the discovery of novel mechanisms of resistance to ET in BC and the identification of promising therapeutic targets have been the focus of BC research in recent years.

Cancer cells are known to have evolved many strategies to evade regulated cell death (RCD) and this resistance to cell death has emerged as one of the hallmarks of tumors [7]. Investigation of these mechanisms is crucial for understanding cancer, and induction of RCD is an important and promising way for cancer therapies. A new cell death mechanism dependent on copper metabolism called cuproptosis is currently being confirmed and relies on mitochondrial respiration [8]. In contrast to other RCDs (apoptosis, ferroptosis, pyroptosis, necroptosis, etc.) that have been extensively studied, the subprogram of cuproptosis differs concerning initial stimuli, intermediate activation events, and end effectors. Intracellular copper accumulation can trigger the aggregation of lipoacylated proteins and subsequent loss of iron and sulfur cluster proteins, resulting in proteotoxic stress and ultimately cell death [9]. RCD is a double-edged sword during tumorigenesis, and selective manipulation of RCD can become a new solution to combat cancer [10]. Ferroptosis, for example, is the research hotspot in RCD just before the report of cuproptosis, which is a form of iron-dependent RCD driven by unrestricted lipid peroxidation. Ferroptosis contributes an important function in inflammation-related immunosuppression within the tumor microenvironment (TME), which provides a link between therapeutic responses and the initiation of various types of cancers [11].

Cuproptosis is a newly entering RCD in the public eye, which has a significant difference from other oxidative stress-related cell death and has generated much interest and potential for the treatment of cancer. In triple-negative BC, it is known that energy production can be reduced by mitochondrial copper depletion via oral administration of the bioavailable copper chelator tetrathiomolybdate, which correlates significantly with a positive effect on patient survival [12–14]. Furthermore, nanoparticles based on copper chelates have also become a hot topic in BC therapeutic research and development [15–17], but few studies have focused on copper homeostasis and response to endocrine therapy. Therefore, elucidation of the possible roles of cuproptosis in the development of resistance to ET in BC will likely yield novel therapeutic avenues in endocrine-resistant BC.

The competing endogenous RNA (ceRNA) hypothesis was a commonly studied model of gene expression regulation, transcripts such as messenger RNA (mRNA) and long-chain noncoding RNA (lncRNA) regulate their expression levels by competing with the same microRNA (miRNA) via miRNA response elements (MREs), thereby affecting the function of cells [18]. miRNAs modulate mRNA abundance by binding to transcripts of target genes, typically inhibiting translation, whereas different RNA molecules can regulate each other indirectly by competing for a shared limited miRNA. This hypothesis predicts that in a ceRNA network, the pattern of miRNA expression should be opposite to that of its target gene mRNA and upstream lncRNA. In contrast, mRNA and lncRNA are expected to have the same expression pattern.

The current study aimed to obtain candidate cuproptosis-related genes (CRGs) associated with resistance to ET by co-analyzing prognostic CRGs in patients with ER+ BC in TCGA-BRCA with differentially expressed CRGs in human-derived ET-sensitive and ET-resistant ER+ BC cell lines. And the specific mechanism of ceRNA network regulation of key CRGs in ET resistance may hold promise in the search for novel therapeutic targets for ET-resistant BC.

## Materials and methods

### Acquiring and preprocessing publicly available data

The RNA-sequencing (including mRNA, lncRNA, miRNA) and clinical data of breast cancer patients in TCGA-BRCA were downloaded from The Cancer Genome Atlas (TCGA, https://portal.gdc.cancer.gov/) database, which contains the expression data of 17,876 protein-coding mRNAs, 12,824 lncRNAs and 1881 miRNAs in 1109 BC tissues and 113 adjacent normal tissues from 1092 cases. Expression data for mRNAs, lncRNAs, and miRNAs from human BC cells, ET-sensitive (MCF-7) and dual tamoxifen and fulvestrant-resistant (LCC9) [19], were obtained from Gene Expression Omnibus (GEO, https://www.ncbi.nlm.nih.gov/geo/) database, with accession number GSE159968 and GSE159979 [20]. To separate mRNAs and lncRNAs from the expression matrix in both TCGA-BRCA and GSE159968, the comprehensive gene annotation was acquired from GENECODE (https://www.gencodegenes.org/human/). The cuproptosis-related genes (*DLAT, PDHA1, LIAS, DLD, DBT, GCSH, DLST, PDHB, SLC31A1, FDX1, LIPT1, ATP7A, ATP7B*) were taken from the study by Tsvetkov et al. [9]. The immunohistochemical (IHC) staining images were retrieved from the Human Protein Atlas (HPA, http://www.proteinatlas.org/). The microarray data from 298 BC patients who underwent 5 years of tamoxifen endocrine therapy and corresponding information on distant recurrent metastases, with GEO accession number GSE17705 [21].

### Establishment of a cuproptosis-related prognostic signature in ER+ BC

An analysis of cuproptosis-related genes (CRGs) with differential expression in TCGA-BRCA among 1109 BC samples and 113 adjacent normal samples was carried out by "limma" package [22] (Version: 3.50.0), false discovery rate (FDR) < 0.05 was considered to be significant. Expression data and survival information for ER+ BC patients were retrieved from TCGA-BRCA based on immunohistochemistry results. Prognostic CRGs in ER+ BC were filtered by undergoing Cox regression and least absolute shrinkage and selection operator (LASSO) regression analyses through the use of “survival” (Version: 3.2-13) and “survminer” (Version: 0.4.9) and “glmnet” [23] (Version: 4.1-3) package, the risk score of each patient was calculated by the formula Risk score = Ʃ (βi × Expi), in which, “βi” represents the LASSO correlation coefficient of gene “i”, while “Expi” represents gene “i”’s expression. The median risk score was used to classify ER+ BC patients into high-risk or low-risk groups. Survival curves were plotted using The Kaplan–Meier (KM) with the “survminer” package, and the log-rank test was used to compare survival among subgroups.

### Immune infiltration, immune function, TME, drug sensitivity, and immunotherapy response analysis

The “e1071” (https://cran.r-project.org/web/packages/e1071/index.html) package (Version: 1.7-9) was loaded as a precondition for “CIBERSORT”. By using the gene expression data of a mixed cell population, CIBERSORT [24] can estimate the abundances of the member cell types based on the expression level of cellular signature genes. While the 22 immune cell gene signature (LM22) was downloaded from the CIBERSORT site (https://cibersort.stanford.edu/), differences in immune cell abundances between high- and low-risk groups were assessed. To compare immune functions among the high- and low-risk groups, the “limma” and “GSEABase” (Version: 1.56.0) packages were used to run single sample Gene Set Enrichment Analysis (ssGSEA). The ssGSEA is an extension of the GSEA method that enables the characterization of cell states based on the activity levels of biological processes and pathways rather than through the expression levels of individual genes, allowing the calculation of immune cell infiltration scores when the set of genes associated with the immune cell marker is used [25]. For quantification of the TME, ESTIMATE score, immune score, stromal score, and tumor purity were accessed by the “estimate” package (https://bioinformatics.mdanderson.org/estimate/rpackage.html). The Estimation of STromal and Immune cells in MAlignant Tumours using Expression data (ESTIMATE) [26] is a method that uses gene expression signatures to infer the ratio of stromal cells and immune cells in tumor samples, that is, the higher the stromal cells and immune cells content, the lower the tumor purity, and vice versa the higher the tumor purity. Using the "limma" package, expression levels of 33 immune checkpoint genes between the high- and low-risk groups were assessed, while Pearson's correlation test was used to analyze the correlation between risk signature genes and immune checkpoint genes. The expression profiles of ER+ BC were uploaded to Tumor Immune Dysfunction and Exclusion website (TIDE, http://tide.dfci.harvard.edu/), to calculate T-cell dysfunction and T-cell exclusion from each sample, and therefore the response to immune checkpoint blockade (ICB) in both high- and low-risk patients could be predicted [27]. The “pRRophetic” [28] package was implemented to calculate half maximal inhibitory concentrations (IC50) and thus predict the chemotherapeutic responses. The main algorithms are based on Geeleher et al. [28] group's 2014 Genome Biology publication, It is possible to predict clinical drug response using baseline levels of gene expression and in vitro drug sensitivity in cell lines. The chemotherapeutic and targeted medicine were then screened by the “limma” package with *P* < 0.001 as the significant difference. To further uncover pathway differences between high- and low-risk groups, “limma” and “GSVA” [29] (Version: 1.42.0) packages were used to proceed with gene set variation analysis (GSVA). GSVA is an unsupervised, parameter-free method for the enrichment of gene sets from both the microarray and RNA-seq data, which can analyze the enrichment of gene sets (pathways) in each sample based on a matrix [29].

### Construction of the cuproptosis-related PPI network and screening for functional core genes

A list of 13 CRGs was uploaded to the STRING website (https://cn.string-db.org/) for analysis of protein interaction (PPI) networks, screened using a combined score > 0.7. Next, the data were imported into Cytoscape (version: 3.7.2), the hub genes were screened by the “cytoHubba” and “NetworkAnalyzer” tools, and the nodes were then ranked according to their degree value. The expression profiles of the 13 CRGs were extracted from GSE159968, differential expression analysis between human BC cells LCC9 and MCF-7 was carried out using the packages “limma” and “edgeR”, genes with an adjust *P* value < 0.05 and |log[fold-change]| (|logFC|) > 0.585 were considered to be differentially expressed genes (DEGs). The intersections of DEGs, signature genes, and hub genes were assumed as the core functional genes.

### Establishment of the cuproptosis-related ceRNA network in ET-resistant BC

To further reveal potential mechanisms of resistance to ET in ER+ BC, a co-expressed regulatory network consisting of cuproptosis-related mRNA, miRNA, and lncRNA was constructed. Presumably, the variable pattern of expression in the mRNA and its upstream miRNA should be opposite, while the variation trend of candidate lncRNA and mRNA must be the same. The upstream miRNAs of cuproptosis-related mRNA were predicted by the intersection of 4 databases (miRDB [30] (http://mirdb.org/), miRWalk (http://mirwalk.umm.uni-heidelberg.de/), RNA22 [31] (https://cm.jefferson.edu/rna22/Interactive/), RNAlnter (http://www.rna-society.org/rnainter/)). For miRNAs differentially expressed (DEMs) in LCC9 and MCF-7 cells, the "edgeR" and "limma" packages were used to filter the data with an adjusted *P* value < 0.05 and |logFC|> 0.585. Candidate miRNAs consisted of the intersection of databases predicted miRNA and DEMs. The lncRNAs were selected by DEGs (mRNA and lncRNA) between LCC9 and MCF-7 cells using “limma” and “edgeR” packages with adjust *P* value < 0.05 and |logFC|> 0.585. The survival-related lncRNAs in ER+ BC from TCGA-BRCA were identified by “survival” and “limma” package using univariate Cox analysis, with a threshold of hazard ratio (HR) > 1 and *P* < 0.05. The candidate lncRNAs were the intersection of DElncRNAs and survival-related lncRNAs.

Pearson's correlation analysis was performed between mRNA-miRNA, miRNA-lncRNA, and mRNA-lncRNA expression separately to improve the accuracy and reliability of this ceRNA network. The correlation in mRNA-lncRNA must be positive with r > 0, while the correlation in mRNA-miRNA and miRNA-lncRNA must be negative with r < 0. Kaplan Meier (K-M) survival curves of the miRNA and lncRNA in the constructed cuproptosis-related ceRNA network were also drawn to validate the prognostic value in ER+ BC patients again.

## Results

### Four CRGs were found to be significantly correlated with ER+ BC prognosis

**Fig. 1:**
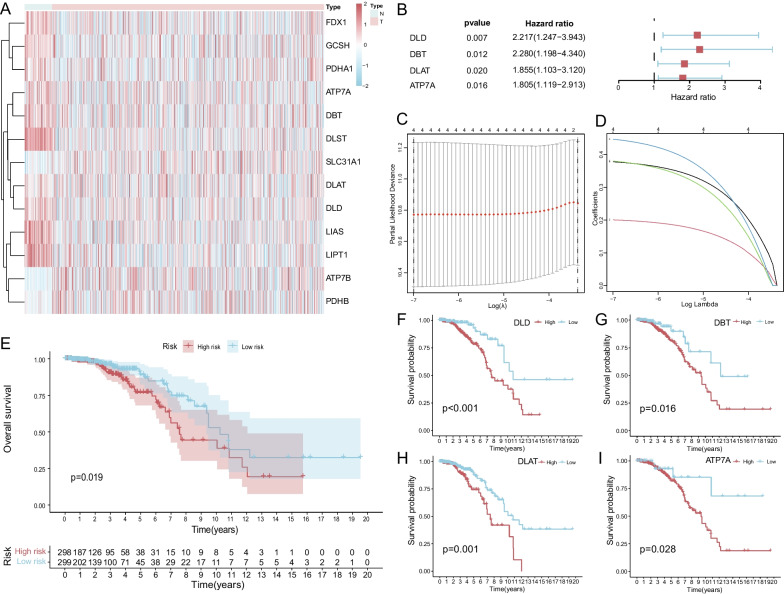
*DLD, DBT, DLAT*, and *ATP7A* were significantly correlated with the prognosis of ER+ BC. **A** Heatmap of 13 CRGs significantly different between BC samples and normal breast tissues; **B** *DLD, DBT, DLAT,* and *ATP7A* were correlated significantly with the prognosis of ER+ BC in univariate Cox regression analysis; **C** LASSO coefficient profiles of the 4-CRGs; **D** Cross-validation for tuning parameter selection in the proportional hazards model; **E** K–M survival curves of high- and low-risk groups; **F**–**I** K–M survival curves according to *DLD, DBT, DLAT, ATP7A* expression level in 597 ER+ BC patients

**Fig. 2:**
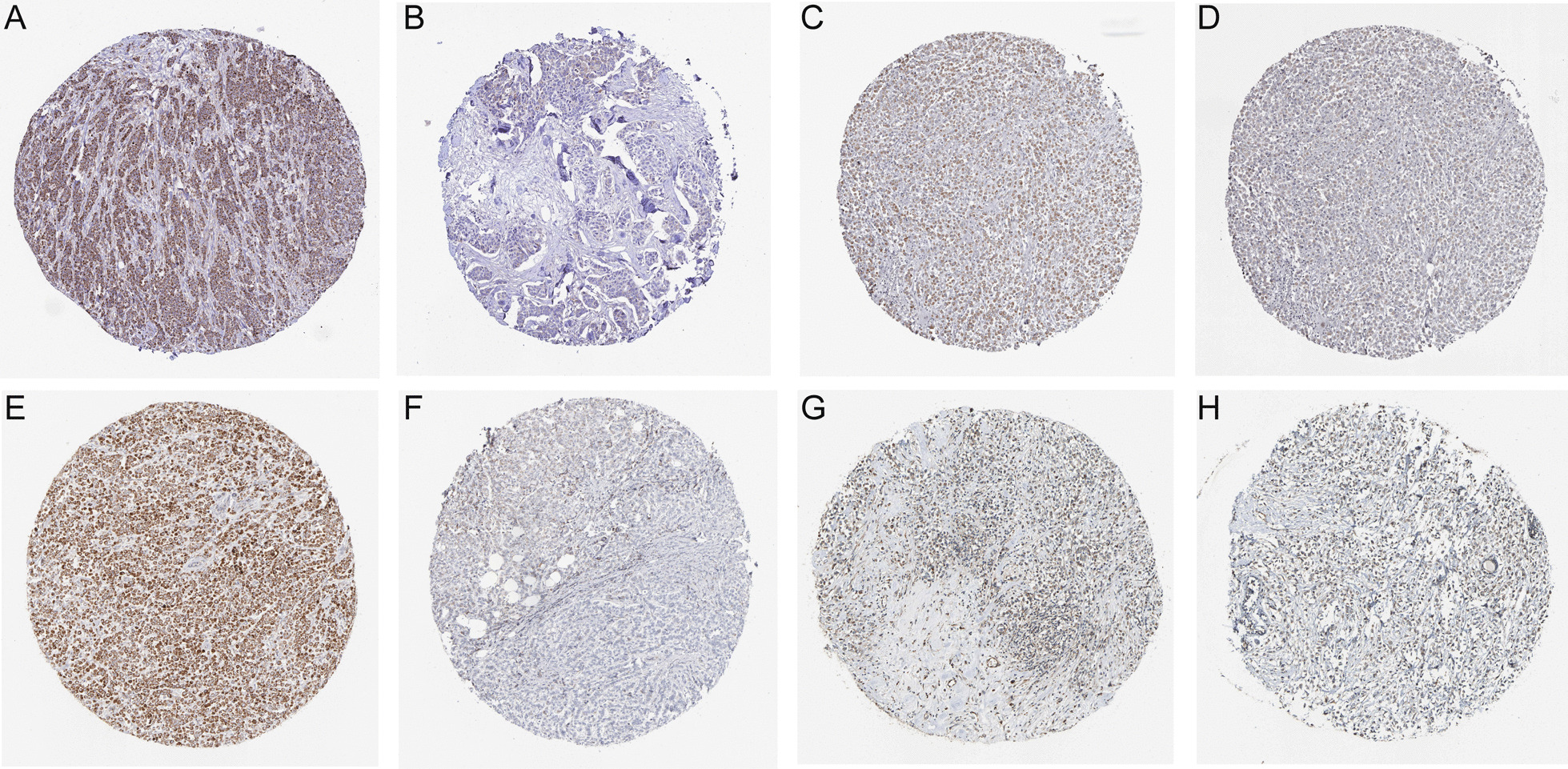
The IHC staining images of 4-CRGs were retrieved from the HPA website. **A** DLD high expression (HPA044849, female, age 40, duct carcinoma, patient id: 2091); **B** DLD low expression (HPA044849, female, age 83, duct carcinoma, patient id: 2160); **C** DBT moderate expression (HPA026485, female, age 61, duct carcinoma, patient id: 1910); **D** DBT low expression (HPA026481, female, age 61, duct carcinoma, patient id: 1910); **E** DLAT high expression (CAB003782, female, age 61, duct carcinoma, patient id: 1910); **F** DLAT low expression (CAB003782, female, age 60, lobular carcinoma, patient id: 2199); **G** ATP7A moderate expression (HPA012887, female, age 61, duct carcinoma, patient id: 1910); **H** ATP7A low expression (HPA012887, female, age 51, lobular carcinoma, patient id: 2083)

Through differential expression analysis of 1109 BC specimens and 113 adjacent normal specimens in the TCGA-BRCA, the mRNA expression levels of 13 CRGs were found to be significantly different (Fig. 1A). Indicating that the extent of copper metabolism in BC cells was markedly changed. Given that TCGA-BRCA clinical information does not include whether the patient is endocrine-resistant, only 597 patients with unequivocal ER+ status immunohistochemistry results were recruited for further analyses. The occurrence of endocrine resistance is known to severely affect the prognosis of patients with ER+ BC, and it is speculated that prognosis-related genes may be linked to the development of ET resistance. Four CRGs (*DLD, DBT, DLAT, and ATP7A*) were found to be significantly associated with prognosis by univariate Cox regression analysis in 597 patients with ER+ BC (Fig. 1B). The four selected CRGs were incorporated into a LASSO regression model (Fig. 1C, D), the formula for the risk signature was defined as follows: Risk score = DLD*0.378 + DBT*0.201 + DLAT*0.380 + ATP7A*0.447 (Table 1). Individual 4-CRG risk scores were calculated for each patient, the 597 patients were then divided into high- and low-risk groups based on the median value of the risk scores. A significant difference in overall survival (OS) was found between the two groups through KM survival analysis (Fig. 1E), which indicated that a lower risk score was associated with improved survival. KM analysis of OS was analyzed according to 4-CRGs expression levels respectively, results demonstrated that low 4-CRGs expression levels were related to better survival trends (Fig. 1 F-I). To determine the expression of 4-CRGs in BC tissues, we obtained the corresponding IHC staining images from the HPA website (Fig. 2A–H), it could be seen that the 4-CRGs are all expressed in BC tissues, different patients with BC could have different levels of staining intensity.

**Table 1:** The correlation coefficient of the 4-CRGs

Gene	Coefficient
*DLD*	0.377517414344967
*DBT*	0.200634717156064
*DLAT*	0.379822335291763
*ATP7A*	0.446528173171558

### Differences in immune infiltration, TME, and responses to therapy between high- and low-risk groups

**Fig. 3:**
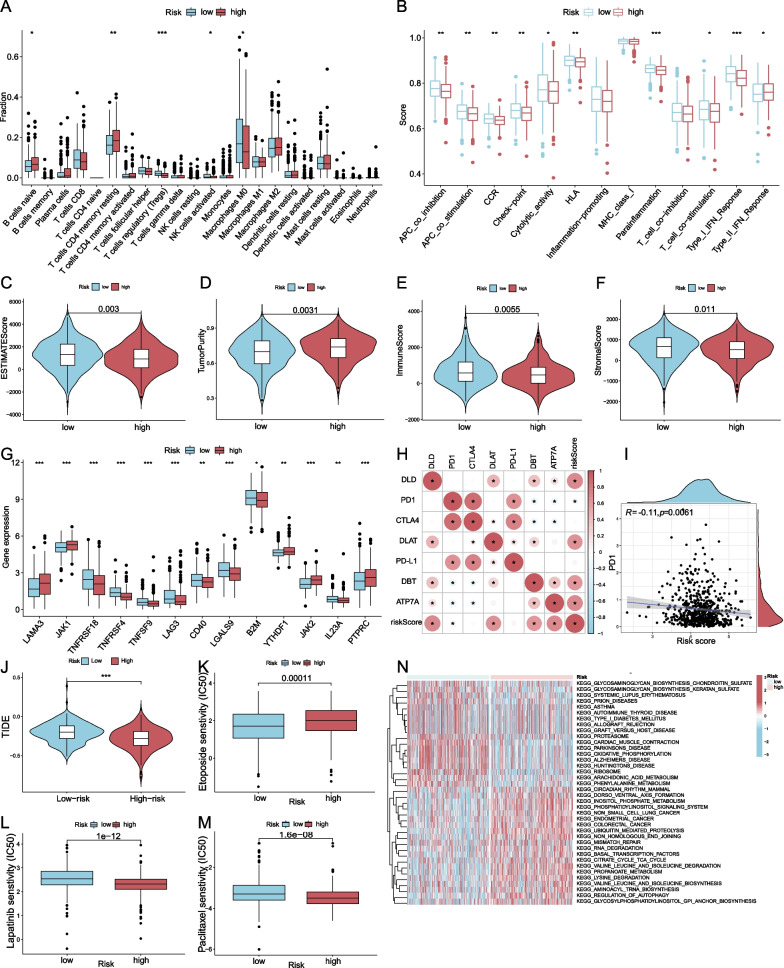
Immune infiltration, TME, and therapy responses analysis. **A** CIBERSORT analysis found the abundances of 5 immune cells were significantly different between the two groups; **B** ssGSEA analysis showed 10 immune functions activated significantly different between the two groups; **C**–**F** Difference in ESTIMATE score, tumor purity, immune score, and stromal score between high- and low-risk group; **G** 13 immune checkpoint genes expressed differently among two groups; **H** The correlation map of the expression levels of 4-CRGs, *CTLA4*, *PD1*, *PD-L1*, and risk score; **I** The expression level of *PD1* is significantly negatively correlated with a risk score, with a coefficient of − 0.11; **J** The difference in immune therapy response predicted by TIDE in high- and low-risk group; **K**–**M** The difference in IC50 values of etoposide, lapatinib, paclitaxel in high- and low-risk group; **N** The heatmap of top 50 differential activated pathways among the high- and low-risk group. (*p < 0.05, **p < 0.01, ***p < 0.001)

Immune infiltration analysis using "CIBERSORT" revealed that abundances of 5 among 22 immune cells were significantly different between the high- and low-risk groups (Fig. 3A). In the high-risk group, there are higher proportions of naïve B cells, T cells CD4 memory resting, and lower proportions of T cells regulatory (Tregs), natural killer (NK) cells activated, macrophages M0. Analysis of immune function using the ssGSEA method revealed that the high-risk group has the relative up-regulated function of type II IFN response and relative down-regulated functions of antigen-presenting cell (APC) co-inhibition, APC co-stimulation, chemokine receptors (CCR), check-point, cytolytic activity, human leukocyte antigen (HLA), Para inflammation, T cell co-stimulation and type I IFN response (Fig. 3B). The “ESTIMATE” package was applied to estimate the fraction of stromal and immune cells in high- and low-risk group microenvironments. The low risk-group has significantly higher proportions of stromal and immune cells, while the high-risk group contained a higher percentage of tumor cells and a higher estimate score (Fig. 3C–F). As immune-checkpoint pathways make considerable contributions as a major mechanism of immune resistance, the expression levels of 38 common checkpoint regulators retrieved from literature (Additional file 1) were compared between high- and low-risk groups. Thirteen immune checkpoint genes were differentially expressed between the two groups (Fig. 3G). The curative effects of immune checkpoint inhibitor (ICI) therapies, primarily *CTLA4*, *PD1*, and *PD-L1*, provide a link to the expression levels of the corresponding signaling molecules in specific tumors and immune cells [32]. The correlation between the mRNA levels of 4-CRGs, *CTLA4*, *PD1*, *PD-L1,* and risk score was calculated (Fig. 3H), it could be seen that *CTLA4* has a significant inverse correlation with *DBT* and *ATP7A*, while *PD1* has a significant negative correlation with *DBT*, *ATP7A*, and the risk score (Fig. 3I). Prediction of response to immunotherapy is based on the TIDE algorithm, which suggests that the low-risk group may benefit more from treatment (Fig. 3J) (Additional file 2). The drug sensitivity analysis using “pRRophetic” demonstrated that the IC50 values of 67 chemotherapeutic drugs were significantly different between high- and low-risk groups, mainly including etoposide, lapatinib, paclitaxel (Fig. 3K–M). The low-risk group is more sensitive to etoposide, while the high-risk group is more sensitive to lapatinib and paclitaxel. Pathway activity was calculated using GSVA, with the top 50 most significant pathways displayed by the heatmap (Fig. 3N). The pathway differences between the high- and low-risk groups indicate the various biological behaviors due to the expression levels of the four cuproptosis-related signature genes diverging. Several cancer-related pathways, such as non-small cell lung cancer, endometrial cancer, and colorectal cancer, have been shown to have significantly higher activity in the high-risk group, leading to the expectation that CRGs expression level has potential value across a wide range of cancer types and appears to have an oncogenic effect.

### *DLD* is the core CRG associated with ET resistance in ER+ BC

**Fig. 4:**
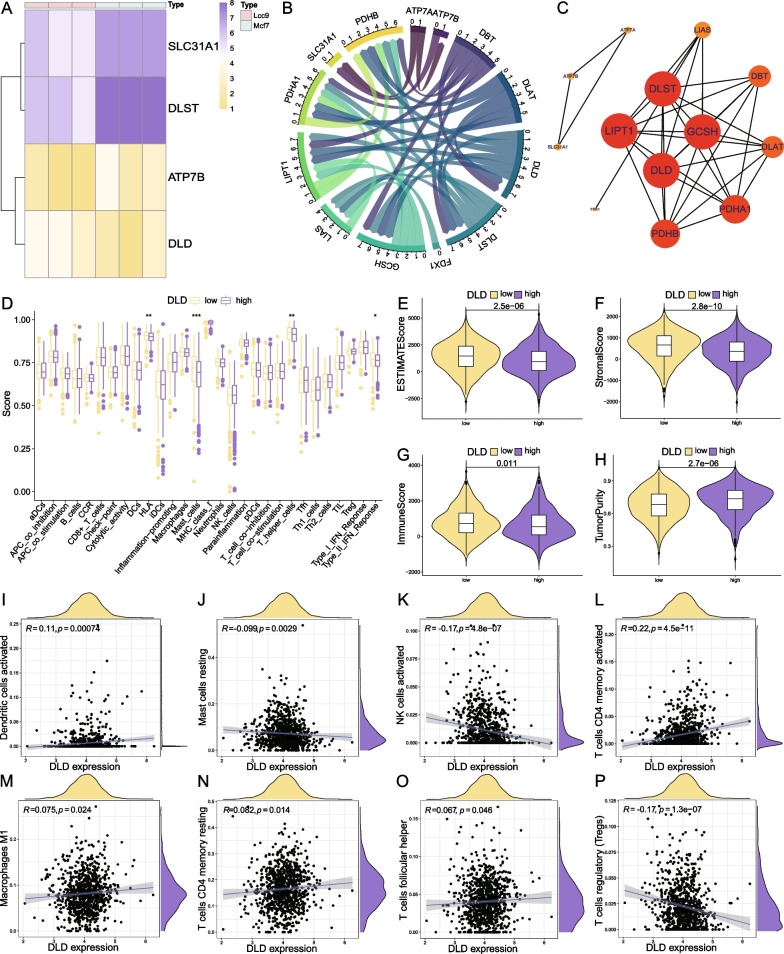
*DLD* is the core CRG associated with ET resistance in ER+ BC. **A** The heatmap of four CRGs with significant differences between LCC9 and MCF-7 cell lines; **B** The chord plot of the interactions between 13 CRGs; **C** The PPI network of 13 CRGs, the sizes, and colors of nodes are defined by the node degree, the larger the node size and the redder the color, the higher the degree; **D** The immune function analysis using ssGSEA found 4 functions significantly different between the high and low expression level of *DLD*; **E**–**H** TME analysis found significant differences between the high and low expression level of *DLD* in ER+ BC; I-P) Scatterplots of the correlations between the expression level of *DLD* and infiltration levels of 8 immune cells, dendritic cells activated (*R* = 0.11), mast cells resting (*R* = − 0.099), NK cells activated (*R* = − 0.17), T cells CD4 memory activated (*R* = 0.22), macrophages M1 (*R* = 0.075), T cells CD4 memory resting (*R* = 0.082), T cells follicular helper (*R* = 0.067), T cells regulatory (*R* = − 0.17). (*p < 0.05, **p < 0.01, ***p < 0.001)

Differential expression analysis between the sequencing data from the human ER+ BC cell line MCF-7 (ET sensitive) and LCC9 (tamoxifen and fulvestrant resistant) revealed four CRGs that were significantly different from the 13 CRGs (Fig. 4A). To build potential protein interactions, the list of 13 CRGs was uploaded to the online tool "String", only edges with a combined score > 0.7 were reserved (Fig. 4B). The PPI data downloaded from String were then imported into Cytoscape software, hub genes were selected using the cytoHubba plugin, and nodes were arranged according to their degree values. *DLD, LIPT1, DLST*, and *GCSH* were found to be the hub genes of the interaction of the cuproptosis-related protein (Fig. 4C). *DLD* was the intersection gene of CRGs related to ER+ BC prognosis (*DLD, DBT, DLAT, ATP7A*), CRGs differentially expressed between LCC9 and MCF-7 cell lines (*SLC31A1, DLST, ATP7B, DLD*), and hub genes from the cuproptosis-related PPI network (*DLD, LIPT1, DLST, GCSH*). Thus, suggesting that *DLD* is the central CRG associated with resistance to ET in ER+ BC. Analysis of immune function by ssGSEA dependent on the expression level of *DLD* discovered that the low expression of the *DLD* group has relatively higher activation functions of HLA, mast cells, T helper cells, and type II IFN response (Fig. 4D). The TME analysis illustrated that the low *DLD* expression is related to higher stromal score, immune score, ESTIMATE score, and a lower percentage of tumor cells (Fig. 4E–H). CIBERSORT immune infiltration analysis found that the expression level of *DLD* is significantly correlated with 8 of the 22 immune cells (Fig. 4I–P). The expression of *DLD* positively correlated with the infiltrating levels of dendritic cells activated (*R* = − 0.099), T cells CD4 memory activated (*R* = 0.22), macrophages M1 (*R* = 0.075), T cells CD4 memory resting (*R* = 0.082), T cells follicular helper (*R* = 0.067) and negatively correlated with mast cells resting (*R* = − 0.099), NK cells activated (*R* = − 0.17), T cells regulatory (*R* = − 0.17).

### Construction of the ceRNA network of cuproptosis-related gene *DLD*

**Fig. 5:**
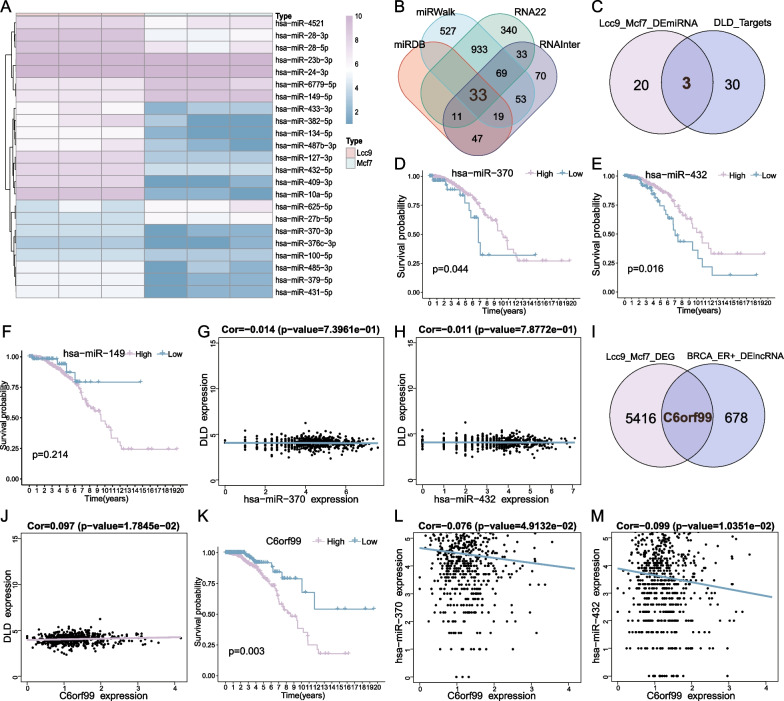
Construction of the ceRNA network of cuproptosis-related gene *DLD*. **A** The heatmap of 23 significantly differentially expressed miRNAs in LCC9 and MCF-7 cell lines; **B** The Venn gram of miRNAs with *DLD* as target gene predicted in four databases; **C** The Venn gram of predicted miRNAs and differential expressed miRNAs; **D** KM survival analysis based on the expression level of hsa-miR-370-3p; **E** KM survival analysis based on the expression level of hsa-miR-432-5p; **F** KM survival analysis based on the expression level of hsa-miR-149-5p; **G**, **H** Scatterplots of the correlations between the expression level of *DLD* and that of hsa-miR-370-3p, hsa-miR-432-5p; **I** The Venn gram of DEGs in LCC9 and MCF-7 cell lines and prognostic lncRNAs in 597 ER+ BC patients; **J** Scatterplot of the correlations between the expression level of *DLD* and that of C6orf99; **K** KM survival analysis based on the expression level of C6orf99; **L**, **M** Scatterplots of the correlations between the expression level of C6orf99 and that of hsa-miR-370-3p, hsa-miR-432-5p

**Fig. 6:**
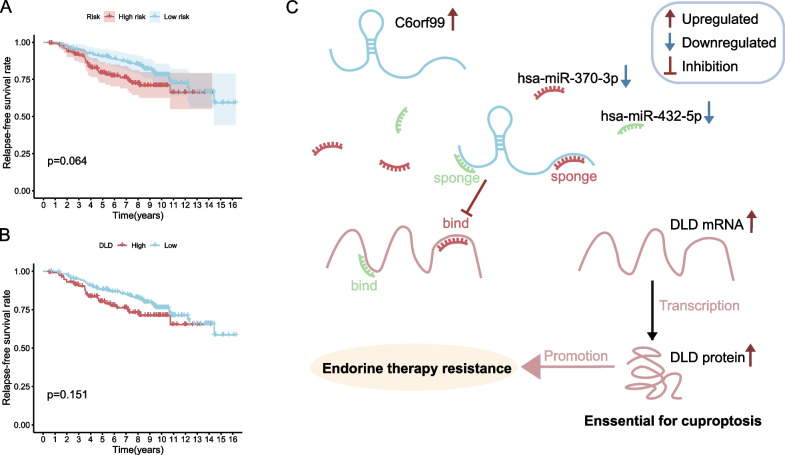
Validation of the CRGs risk model. **A** The KM plot of RFS based on CRGs risk model; **B** The KM plot of RFS based on DLD expression level; **C** Mechanistic hypothesis map for the cuproptosis-related ceRNA network in endocrine therapy-resistant ER+ BC

After *DLD* was identified as the core CRG in ER+ ET resistance BC, to find the upstream miRNAs in the *DLD* ceRNA network, the expression levels of 23 miRNAs were found to be significantly different between the miRNA sequencing data of LCC9 and MCF-7 (Fig. 5A). Subsequently, the upstream regulatory miRNAs of *DLD* were predicted by the miRDB database, miRWalk database, RNA22 database, and RNAlnter database, 33 miRNAs were the intersection of prediction results of 4 databases (Fig. 5B). The 23 differential expressed miRNAs between LCC9 and MCF-7 were then intersected with the 33 predicted miRNAs from 4 databases, 3 miRNAs were found in common (Fig. 5C). KM survival analysis was performed on 597 ER+ BC patients based on the expression levels of 3 miRNAs, respectively (Fig. 5D–F), high expression levels of hsa-miR-370-3p and hsa-miR-432-5p were related to better prognosis while the high expression level of hsa-miR-149-5p was related with worse prognosis. This could be seen in Fig. 1F, the high level of *DLD* expression correlated with a poorer prognosis. The correlation between 3 miRNAs and *DLD* is expected to be negative depending on the mechanism of action of the ceRNA network. Thus, the actual trend of hsa-miR-370-3p and hsa-miR-432-5p was in agreement with the theoretical prediction. In addition, Pearson's method was applied to analyze the correlation between the expression level of the DLD and that of the hsa-miR-370-3p and hsa-miR-432-5p (Fig. 5G, H), both miRNAs were negatively correlated with DLD mRNA (*R* = − 0.014 and *R* = − 0.011), but, unfortunately, the *P*-values were greater than 0.05, this may due to the variable of miRNA stability variable and the few mechanisms understood [33]. A total of 679 lncRNA were found to have prognostic value using univariate Cox analysis in 579 ER+ BC, whereas 5417 mRNA and lncRNA were found to be significantly differentially expressed in LCC9 and MCF-7 cell lines, only one common lncRNA C6orf99 was obtained by taking both sets to be the intersection (Fig. 5I). Pearson’s correlation analysis indicated that the expression level of C6orf99 was positively related to the expression level of *DLD* mRNA (*R* = 0.097, *P* < 0.05) (Fig. 5J), negatively related to the expression levels of hsa-miR-370-3p (*R* = − 0.076, *P* < 0.05) (Fig. 5L) and hsa-miR-432-5p (*R* = − 0.099, *P* < 0.05) (Fig. 5M). Moreover, KM survival analysis showed that the low expression level of C6orf99 has a better prognosis (Fig. 5K), this trend was the same as that observed in *DLD* previously. Based on the foregoing results, a cuproptosis-related ceRNA network of ET resistance in ER+ BC was ultimately established, which implicates a possible mechanism in endocrine therapy resistance (Fig. 6).

To further validate the risk model of CRGs constructed based on data from ER+ BC patients in TCGA-BRCA, the microarray data from 298 breast cancer patients who underwent 5 years of tamoxifen endocrine therapy and corresponding information on distant recurrent metastases were analyzed [21]. The Relapse-free survival (RFS) rate was assessed by the KM curve (Fig. 6A), although the *p*-value is not statistically significant, the non-recurrence rate for the low-risk score is higher in the interval of 2–10 years than in the high-risk group. The definition of intrinsic/acquired ET resistance was first clarified at the European society for medical oncology (ESMO) in 2014 [34]. Where intrinsic ET resistance was defined as relapse within 2 years during adjuvant ET or progression within 6 months of MBC first-line ET, while acquired ET resistance was defined as relapse after 2 years during adjuvant ET or relapse within 12 months after completion of adjuvant ET or progression ≥ 6 months after the start of MBC first-line ET [35]. In other words, for patients in this dataset, relapses within 6 years are due to tamoxifen resistance, and relapses after 6 years should be tamoxifen-sensitive relapses. As can be seen from the figure, the CRGs-based risk score was better at distinguishing between high- and low-risk patients within 6 years, but the relapse rates in the two groups tended to be close after 10 years, suggesting that the score may be applicable only for predicting the risk of ET resistance relapse but not for the risk of relapse in ET-sensitive patients. Interestingly, when KM curves for RFS rates were plotted for these patients based on *DLD* expression levels (Fig. 6B), they showed a very similar trend to the risk score, suggesting that *DLD* may play a central role in the CRGs of this risk model. The mechanistic hypothesis map for the cuproptosis-related ceRNA network in ET-resistant ER+ BC was shown in Fig. 6C.

## Discussion

More recent research suggests that cuproptosis is a novel form of copper-induced mitochondrial cell death via the targeting of lipoylated proteins of the tricarboxylic acid (TCA) cycle, which successively leads to aggregation of lipoylated proteins, loss of iron-sulfur cluster proteins, proteotoxic stress and eventually cell death [9]. The mitochondria are not only responsible for cuproptosis but are also multifaceted regulators of cell death such as apoptosis and ferroptosis [8]. It has previously been found that mitochondrial stress adaption can excite aromatase inhibitor (AI) ET resistance in human BC cells [36], in addition, mitochondrial ER alternation can promote further resistance to ET [37]. On the other hand, copper transport systems are essential for intracellular transport and processing of cisplatin, indicating its non-negligible role in triggering cisplatin efficacy [38]. These data not only motivate the idea that mitochondrial stress may be the fundamental molecular mechanism of metal-induced toxicity but also provide a clue that copper accumulation may be linked to drug sensitivity.

There have been several studies on bioinformatics analysis of cuproptosis in BC, Li et al. [39] analyzed the prognostic role of CRGs in all BCs, while Sha et al. [40] and Cheng et al. [41] conducted on triple-negative BC. Besides, Li et al. [42] and Li et al. [43] come to similar conclusions that SLC31A1 is associated with poor prognosis of BC. In our study, SLC31A1 also has a higher expression level in the LCC9 cell line but not significantly correlated with prognosis in ER+ BC patients. These authors have obtained interesting results and, to some extent, have demonstrated the value of cuproptosis in BC. However, few studies have been done for ER+ BC. Breast cancer is highly heterogeneous, and different molecular typing is used for different treatment strategies. As the most prevalent subtype, ET is the pivotal treatment for ER+ BC. Therefore, uncovering the mechanism and role of cuproptosis in ET-resistant BC can contribute to the search for new therapeutic strategies.

In this study, the potential associations of cuproptosis and ET resistance in ER+ BC were investigated and a 4-CRGs risk signature consisting of *DLD, DBT, DLAT*, and *ATP7A* was constructed. Among these, dihydrolipoamide dehydrogenase (*DLD*) is a gene that encodes a component of the lipoic acid pathway and proved to be essential for cuproptosis [9]. The study also suggests that *DLD* has strongly implicated in cystine deprivation-induced ferroptosis by causing iron accumulation in mitochondria in head and neck cancer [44]. In melanoma cells, it has been proved that the downregulation of *DLD* can alternate the energy metabolism of mitochondria through decreasing downstream metabolites of the TCA cycle, therefore inducing death via autophagy [45]. The present study found that the higher expression level of *DLD* is associated with poorer clinical outcomes in ER+ BC patients while the dual tamoxifen and fulvestrant-resistant human BC cell line LCC9 also has a significantly higher expressed *DLD* level compared to MCF-7. Based on this evidence, it is hypothesized that the high level of *DLD* expression may protect BC cells from mitochondrial stress-induced death and thereby induce resistance to ET.

Dihydrolipoamide branched chain transacylase E2 (*DBT*), as well as dihydrolipoamide S-acetyltransferase (*DLAT*), are two of the only four enzymes where protein lipoylation can occur, the metabolic complexes of which can regulate carbon entry points to the TCA cycle [9]. The accumulation of copper can increase the lipoylation of mitochondria protein, in addition to this, *DLAT* can be bounded to copper directly and facilitate the aggregation of lipoylated *DLAT* depending on disulfide bonding [8]. Additional studies have also implicated that the variation of *DLAT* is significantly correlated with obesity in humans [46]. Obesity was found to be strongly associated with the occurrence of up to thirteen cancer types, especially ER+ BC in postmenopausal women, factors related to obesity modulate the metabolic signaling pathways in both BC cells and TME, which can be regarded as a molecular link between obesity and BC [47]. In this study, the low expression levels of *DBT* and *DLAT* are protective factors of ER+ BC. With current information, it can be inferred that the dysregulation of protein lipoylation on *DBT* and *DLAT* due to aberrant copper accumulation can affect the TCA cycle, at the same time, the dysregulation expression of obesity-related gene *DLAT* influences the energy metabolic in *ER+ *BC cells and microenvironment, ultimately affecting the responses to specific drugs and BC prognosis.

ATPase copper transporting α (*ATP7A*) has been under wild-type investigation for decades. It has an expression in most tissues and involves in many physiological processes, one of the major functions of this Cu-ATPase is the maintenance of copper homeostasis within the cell through transporting copper across cellular membranes from the cytosol, the dysfunction of *ATP7A* is often associated with severe metabolic dysregulations [48]. On one hand, platinum anticancer drugs such as cisplatin and an oxaliplatin analog, specifically interfere with Cu homeostasis by inhibiting copper transport with Cu-ATPases as a mechanistic and structural basis [49]. On the other hand, the platinum drugs transmembrane translocate in an ATP-dependent way which is similar to that of copper, and the up-regulation of *ATP7A* has been proved to be associated with enhanced platinum drug resistance [50]. Beyond this, *ATP7A* is also described to be involved in autophagy, and vascular endothelial growth factor receptor 2 (*VEGFR2*) degradation in endothelial cells, the loss of *ATP7A* inhibits angiogenic responses via *VEGFR2* signaling [51], while the tumor-stimulated neovascularization is considered as a key step during tumor progression. Others have found that *ATP7A* in adipose tissues has a nonnegligible role in the regulation of aging-related metabolic disease and whole-body fat homeostasis [52]. In this study, we note that the low level of *ATP7A* expression is relevant to a better prognosis in ER+ BC since the precise mechanisms remain speculative but the downstream consequence of perturbation of copper homeostasis may play an essential role.

To probe the possible mechanisms of how the 4-CRGs function in ER+ BC, further analyses focused on risk score based on the expression level of comprehensive 4-CRGs signature. The results display significant differences in immune functions, immune infiltration, and TME by cuproptosis-related risk score, suggesting that the phenotypes of copper metabolism serve important roles in multiple biological processes of hormone-sensitive BC. For example, the CIBERSORT analysis shows that the high-risk group has a lower proportion of NK cells activated, a cell that has been particularly known for its innate ability to recognize and spontaneously kill tumor cells in the field of oncology [53]. Therefore, the lower proportion of NK cells activated may promote tumor immune escape in the tumor immune microenvironment. Interestingly, the high-risk group also owns a lower proportion of Tregs. It is known that Tregs contribute to the suppression of excessive immune activation and coordinate tumor immune evasion as immunosuppressor cells, and have been considered a target of systemic immunotherapies [54]. Similar results which may seem contradictory are observed in immune function analysis too, for instance, both mutually antagonistic immune functions APC co-inhibition and APC co-stimulation are up-regulated in a low-risk group. However, the final immunity state of TME, the main battleground of cancer cells and immune cells, is mainly governed by the delicate and dynamic equilibrium between immune-suppressive and immune-stimulatory mechanisms, metabolic reprogramming in the TME is essential for cancer progression as well as effective immune responses [55]. Leading us to speculate that upregulation of these 4-CRGs may influence intracellular copper concentrations, thereby leading to the reprogramming of lipid metabolism in concert with mitochondrial stress, ultimately altering the immune status of the TME.

Followed by findings that *DLD* is rather prominent both in clinical patients and in cell lines in vitro. Not only is *DLD* significantly related to prognosis in ER+ BC patients, but there is also a significant difference in the expression of dual tamoxifen and fulvestrant-resistant LCC9 cell lines and MCF-7 cell lines sensitive to ET. Furthermore, the PPI network analysis showed DLD to be one of the top-ranked hub genes among 13 cuproptosis-related genes. Subsequent analyses revealed that differences in the levels of *DLD* may also alter immune functions and tumor immune microenvironment, in addition to this, the level of *DLD* expression is statistically significantly correlated with 8 types of immune cells. Then one might speculate that the high level of *DLD* expression may affect the disruption of copper metabolic homeostasis, leading to alternating immune infiltrate and functional status within the TME and varying endocrine therapeutic responses, thus contributing to poor prognosis in ER+ patients.

Together, these findings suggest that *DLD* represents a theoretically potential therapeutic target in ET-resistant BC. Characterization of the ceRNA network linked to *DLD* is critical for exploring the upstream regulatory mechanisms of *DLD* at the level of transcription. Two miRNAs hsa-miR-370-3p and hsa-miR-432-5p are considered to target the mRNA for DLD: first, they are the intersection of four databases of miRNA predictions; second, their expression levels are markedly different between the LCC9 and MCF-7 cell lines; third, they were significantly expressed correlated with survival in ER+ patients from TCGA-BRCA and trended in the opposite direction with DLD. It is to be regretted that the correlation analysis of these two miRNAs and DLD mRNA is not statistically significant, although the coefficients are negative. Perhaps because of the sophisticated interplay and regulation between RNAs, it is known that one lncRNA can regulate several miRNAs simultaneously, and one miRNA can target multiple genes [56], the influence of a single miRNA on certain target gene or ceRNA network may be limited to some extent [57]. The analysis results of lncRNA C6orf99 are relatively ideal, the expression level of which not only correlated significantly with the prognosis of ER+ patients and has a significant difference in LCC9 and MCF-7 cell lines but also coincides with the theoretical trends (a positive correlation with *DLD* mRNA and negative correlation with two miRNAs), the results are statistically significant. There is currently little data on C6orf99, one study found its prognostic value in BC [58], with the other study reporting its potential role in male infertility [59]. There is currently a lack of studies specifically assessing C6orf99 function, this research reports that C6orf99 may involve a cuproptosis-related ceRNA network in patients with ER+ BC and its level of expression may be linked to resistance to ET.

There are some deficiencies and possible limitations to this study. All the data used for preliminary prognosis analysis are publically available datasets, the 4-CRGs risk signature is only a theoretical model, few suitable data from ET-resistant BC patients as an external validation set, the results of the preliminary validation were not very satisfactory, but they can still be illustrative to some extent. It still needs to be validated by further clinical studies to become a predictive model with clinical value. Although *DLD* is an attractive target, its detailed role in ER+ BC has not been validated either in vitro or in vivo, further illustration is required to elucidate the detailed mechanism. This study was the first to report the potential of C6orf99 as an upstream regulator of cuproptosis through the ceRNA network, however, there are few studies of this lncRNA, and further research is required to clarify the specific function of C6orf99 in the human body.

## Conclusion

This study aimed to construct a prognostic model based on the cuproptosis-related genes in ER+ BC with the formula defined as risk score = DLD*0.378 + DBT*0.201 + DLAT*0.380 + ATP7A*0.447. The cuproptosis-related gene *DLD* was considered to be the core gene associated with ET resistance in ER+ BC. In the search for promising therapeutic targets, a ceRNA network consisting of C6orf99/hsa-miR-370-3p and hsa-miR-432-5p/*DLD* was established. These findings identify a copper-metabolism-ET-resistant axis with the potential value for an in-depth study on the prevention and reversal of BC ET resistance.

## Supplementary Information

**Additional file 1.** The gene list of 38 common checkpoint regulators retrieved from literature.

**Additional file 2.** The immunotherapy response prediction results of all the BC samples in TCGA-BRCA based on TIDE algorithm.

## Data Availability

The datasets analyzed during the current study are available in the TCGA-BRCA repository (https://portal.gdc.cancer.gov/projects/TCGA-BRCA) and the GEO database (https://www.ncbi.nlm.nih.gov/geo/). The accession numbers of GEO datasets are GSE159968, GSE159979, and GSE17705.

## Abbreviations

BC: Breast cancer
ER: Estrogen receptor
TAM: Tamoxifen
SERDs: Selective estrogen receptor down-regulators
AIs: Aromatase inhibitors
SERMs: Selective estrogen receptor modulators
RCD: Regulated cell death
TME: Tumor microenvironment
CRGs: Cuproptosis-related genes
ssGSEA: Single sample gene set enrichment analysis
GSVA: Gene set variation analysis
IHC: Immunohistochemical
K–M: Kaplan Meier
TCA: Tricarboxylic acid
*DLD*: Dihydrolipoamide dehydrogenase
*DBT*: Dihydrolipoamide branched chain transacylase E2
*DLAT*: Dihydrolipoamide S-acetyltransferase
*ATP7A*: ATPase copper transporting α
